# Substrate texture properties induce triatomine probing on bitten warm surfaces

**DOI:** 10.1186/1756-3305-4-111

**Published:** 2011-06-17

**Authors:** Raquel A Ferreira, Marcos H Pereira, Marcelo G Lorenzo

**Affiliations:** 1 Escola de Saúde Pública de Minas Gerais, Avenida Augusto de Lima, 2061, Barro Preto, 30190-002, Belo Horizonte, MG, Brazil; 2 Laboratório de Fisiologia de Insetos Hematófagos, Universidade Federal de Minas Gerais. Belo Horizonte, Brazil; 3 Laboratório de Triatomíneos e Epidemiologia da Doença de Chagas, Centro de Pesquisas René Rachou, Avenida Augusto de Lima, 1715, Barro Preto, 30190-002, Belo Horizonte, MG, Brazil

## Abstract

**Background:**

In this work we initially evaluated whether the biting process of *Rhodnius prolixus *relies on the detection of mechanical properties of the substrate. A linear thermal source was used to simulate the presence of a blood vessel under the skin of a host. This apparatus consisted of an aluminium plate and a nickel-chrome wire, both thermostatized and presented at 33 and 36°C, respectively. To evaluate whether mechanical properties of the substrate affect the biting behaviour of bugs, this apparatus was covered by a latex membrane. Additionally, we evaluated whether the expression of probing depends on the integration of bilateral thermal inputs from the antennae.

**Results:**

The presence of a latex cover on a thermal source induced a change in the biting pattern shown by bugs. In fact, with latex covered sources it was possible to observe long bites that were never performed in response to warm metal surfaces. The total number of bites was higher in intact versus unilaterally antennectomized insects. These bites were significantly longer in intact than in unilaterally antennectomized insects.

**Conclusions:**

Our results suggest that substrate recognition by simultaneous input through thermal and mechanical modalities is required for triggering maxillary probing activity.

## Background

Triatomine bugs are vectors of Chagas' disease [1-3], which is caused by the protozoan parasite *Trypanosoma cruzi*. This disease is one of the main public health problems in Latin America. Endemic to 21 countries, the disease affects approximately 15-16 million people, while another 75-90 million are exposed to risk of transmission [4]. In Venezuela, Colombia and some parts of Central America, *Rhodnius prolixus *Stål (1859) is the main vector of this parasite [5].

Triatomine bugs rely on a number of sensory cues that are emitted by their hosts, mainly chemical and thermal, for orienting when they search for a blood-meal. The heat emitted by warm blooded animals is the main cue used by triatomine bugs to locate a host at short distances [6,7]. Indeed, it is the only stimulus that can trigger biting in these bugs [8-10]. A sequence of behavioural responses is shown by *R. prolixus *when confronted with a heat source [8]. Initially, this involves only antennal movements that are followed soon after by locomotor activity during which the antennae are rhythmically flexed up and down (8). Finally, insects extend their proboscis just before they touch the source with one or both antennae [8]. This process of distance estimation is presumably mediated by triangulation based on the integration of the information obtained by both antennae [6]. More recently, it was suggested that thermal gradients formed along the antennae may allow insects to detect nearby heat sources [11]. The antennae of triatomines have a cave organ that is apparently involved in the detection of heat sources [12,13].

Triatomines can discriminate between heat sources of different temperatures [6] and determine the spatial position of these sources at a distance of a few centimetres by using their antennae [10]. Furthermore, it has also been shown that unilaterally antennectomized insects fail to aim their proboscises accurately under the same experimental paradigm [10], evincing that the bilateral integration of antennal inputs is required for this task.

Triatomines obtain their blood meals directly from venules and arterioles [14].

After piercing the host skin, their mouthparts perform sounding movements inside the dermal tissue in order to contact a blood vessel and penetrate into it. This probing activity represents only 6% of the total time they contact the host during feeding [15]. It is a critical phase in the feeding process because the insertion of the mouthparts and their movements under the skin cause tissue damage and induce the release of chemicals that can trigger nerve responses, haemostasis and inflammatory reactions [16]. In fact, *R. prolixus *can locate a blood vessel before establishing contact between its proboscis and the host skin and it has been demonstrated that the thermal sense is also involved in this process [17].

The present study analyses whether different substrate textures induce changes in the biting pattern shown by *R. prolixus*. During our study, we observed that the insects eventually performed prolonged bites on latex covered thermal sources. Consequently, we analyzed the duration of the long bites and whether these tended to occur on the region over the linear heat source. Finally, we evaluated whether the bilateral inputs of both antennae are necessary for the expression of long bites.

## Materials and Methods

### Animals

The insects used for the experiments came from a colony maintained at our institute, and reared under a 12:12 L/D illumination regime, 27 ± 2°C and 60 ± 10% RH. Colony insects were fed exclusively on hen blood, adjusting the protocol according to institutional animal welfare regulations (FIOCRUZ Committee on Animal Welfare license L-058/08). For the experiments, 4^th ^instar bugs were fed weekly on hens until their ecdysis. Fifth-instar *R. prolixus *larvae starved for 30-45 days after moulting were used throughout the experiments. Insects had their eyes covered with black acrylic paint 24 h before the assays in order to prevent them from using visual cues during experiments.

### Metal thermal sources

The thermal setup consisted of a flat aluminium plate that acted as a warm background and a nickel-chrome wire that functioned as a warmer linear heat source. The wire (300 μm thickness) was fastened on the aluminium plate and both were independently thermostated at specific temperatures (accuracy: ± 1°C). These temperatures were measured for verification before and after each trial using a contact thermometer (TES 1300 Taiwan, accuracy: 0.1°C). In most tests, the thermal setup was covered by a latex film to evaluate whether the texture of the substratum altered biting behaviour. In these tests the temperature of the thermal setup was measured and calibrated after the addition of the latex film in order to correct any modification caused by the latex itself.

### Experimental arena

Insects were individually placed into a receptacle located at one end of a rectangular acrylic box (24 × 16 × 8 cm) and released after 10 minutes. A 2 cm wide square opening located in a central position on the base of the box and 10 cm away from the receptacle allowed the insects to contact the thermal setup. The linear heat source was presented in a central position in the opening and parallel to the longitudinal axis of the arena.

### Experimental treatments

For analysing whether insects evaluate the mechanical properties of a substratum after making physical contact with their proboscis, we performed the following assays: first, we developed tests (7 insects) with the metal heat source [17] presented without a latex cover (linear source/no latex). Afterwards, the same heat source was presented with a latex cover to determine whether this induced a change in the biting pattern shown by bugs (14 insects). In both cases, the linear heat source was presented at 36°C and the flat aluminium background at 33°C (linear source/latex). For its corresponding control, the same heat source setup was covered by a latex film and presented to the bugs with the linear heat source and background both at 33°C (14 insects) (flat source/latex). Finally, unilaterally antennectomized insects (15) were tested to evaluate whether the bilateral integration of information detected by both antennae is necessary for the expression of long bites observed with latex covered heat sources. For these assays, the linear heat source (36°) was also presented warmer than the background (33°), and both were covered by a latex membrane (antenectomized/latex). Insects were unilaterally antennectomized 24 h before the onset of assays.

### Data recording and analysis

A video camera sensitive to infrared (IR) light (of a wavelength which is not perceived by these insects [18]) was used to record their behaviour. The camera was mounted over the arena at a zenithal position in relation to the thermal source. The whole experimental setup was illuminated by the built-in infrared LEDs on the video camera.

The behaviour of the insects in the 2 × 2 cm base opening where the thermal setup was presented was videotaped and subsequently analysed. The biting activity of each bug was analyzed via play back of the video record on a TV monitor for an interval of ten minutes that started with the first bite.

The number of proboscis contacts performed by insects on the thermal setup was recorded for comparison between the different treatments. In addition, the number of long bites, their spatial distribution and their duration were also recorded for all treatments. The duration of long bites was measured by means of a chronometer using the playback function of the video player. To characterize the distribution of long bites, the distance between each bite and the linear heat source was measured with 1 mm accuracy. The position of each long bite performed by an insect during the ten minute test was recorded. In order to calculate the proportion of long bites presented by each bug in the different experimental treatments, the number of long bites was divided by the total number of contacts. Based on these data, a mean distance of long bites was calculated for each insect tested. The mean distance of all long bites performed on experiments testing a warmer linear heat source was computed and statistically compared to that observed when the linear and flat sources were presented at the same temperature.

**Figure 1:**
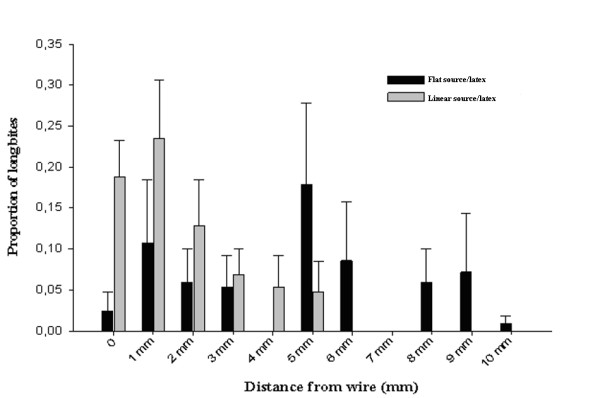
**Distribution of long bites in relation to the linear heat source**. The wire and background were presented at 33°C in the flat source/latex experiment. The linear heat source was presented at 36°C and the background plate at 33°C linear source/latex.

For each insect tested, a proportion of long bites was calculated for each distance category. Afterwards, a mean proportion was computed for each distance category to compare the proportion of long bites performed in the absence or presence of a warmer linear heat source. These results were plotted in Figure 1.

### Statistics

The following parameters were recorded and evaluated: number of bites, mean proportion of long bites, mean duration of long bites and distribution of long bites in relation to the linear heat source. A mean value per insect was computed for each parameter analysed. These means were compared only between treatments (2-sample comparisons): a) intact bugs presented to warmer linear heat sources and heat sources presented at the background temperature, b) intact insects and unilaterally antennectomized insects (both with the temperature of linear source warmer than background). Data analysed in this work for statistical purposes did not meet normality assumptions. Therefore, a Mann-Whitney test was used to compare the results of the different treatments with a confidence interval of 95%.

## Results

All proboscis contacts recorded with the thermal setup presented without latex cover were shorter than 1.5 s. However, in the presence of a latex covered heat source, insects displayed two different biting patterns: 1) short (i.e. <1.5 s) and, 2) long (≥ 1.5 s) proboscis contacts with the source. The complete sequence of penetration and retreat through a membrane by maxillae takes approximately 2 seconds in *R. prolixus *[19]. Therefore, we considered long bites as an indication that probing movements were being performed by bugs. Generally, each insect presented both biting patterns alternatively after contacting latex covered thermal sources. The first pattern (i.e. short contacts) was observed in all experimental treatments and was the most frequent. During long proboscis contacts, insects held the tip of their proboscis on a fixed position over the thermal setup for prolonged time spans. Throughout these intervals, their antennae always remained immobile; subsequent antennal movement returned only a few seconds before proboscis contact with the latex film was interrupted.

**Figure 2:**
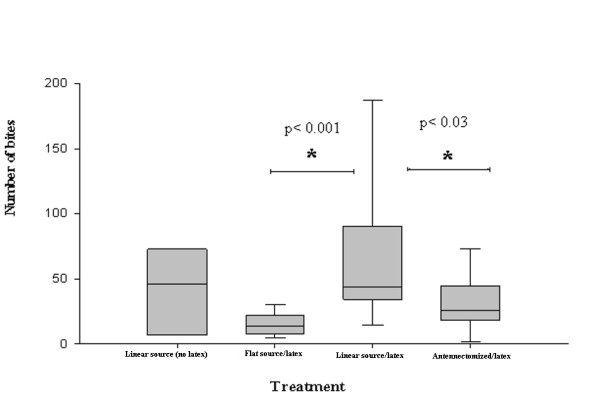
**Number of bites performed by insects**. The wire and background were presented at 33°C in the flat source/latex experiment. The linear heat source was presented at 36°C and the background plate at 33°C in the linear source (no latex), linear source/latex, and antennectomized/latex experiments.

A significantly higher total number of proboscis contacts with the thermal source was observed when the linear heat source was presented at a temperature warmer than the background, compared to the series with both heat sources presented at 33°C (Figure 2, Mann Whitney, p < 0.001). Similarly, intact insects presented a significantly higher total number of contacts than unilaterally antennectomized ones (Figure 2, Mann Whitney, p < 0.03).

The maximum duration of a long proboscis contact on latex covered heat sources was 51 s. After covering the heat sources with a latex membrane, 90% of the proboscis contacts were shorter than 1.5 s. Similarly, the short contacts were 91% of the total contacts in the corresponding control assays (i.e. with wire and plate at 33°C).

**Figure 3:**
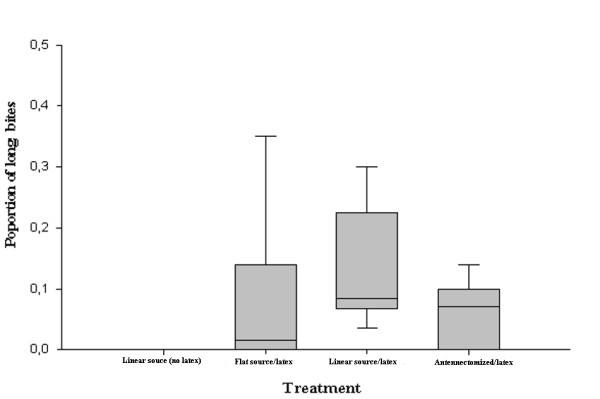
**Mean proportion of long bites**. The wire and background were presented at 33°C in the flat source/latex experiment. The linear heat source was presented at 36°C and the background plate at 33°C in the linear source (no latex), linear source/latex and antennectomized/latex experiments.

Figure 3 shows that insects did not perform long bites when exposed to metal heat sources, in clear contrast to the number of long bites presented by insects confronted with latex covered sources (Figure 3). The absolute number of long bites performed by the insects was higher when the linear heat source presented was warmer than the background (Table 1). Furthermore, an increase in the proportion of long bites was observed, but this was not significant compared to the proportion recorded with homogeneously heated sources (Figure 3, Mann-Whitney, n.s). Additionally, the proportion of long bites presented by intact or unilaterally antennectomized bugs showed no significant difference (Figure 3, Mann-Whitney, n.s).

**Table 1:** The absolute number of long bites performed by the insects

Flat source/latex	Total bites	Long bites	Linear source/latex	Total bites	Long bites	Antennectomized/latex	Total bites	Long bites
1	9	0	1	70	13	1	30	2
2	5	0	2	76	12	2	27	0
3	9	4	3	28	4	3	2	0
4	13	4	4	11	4	4	20	3
5	21	4	5	49	4	5	22	3
6	9	1	6	145	7	6	67	0
7	5	0	7	37	5	7	16	1
8	14	0	8	39	4	8	26	2
9	34	1	9	131	10	9	20	0
10	5	0	10	39	10	10	45	4
11	15	0	11	16	1	11	18	2
12	13	1	12	36	8	12	1	0
13	24	3	13	55	4	13	37	5
14	21	0	14	230	5	14	82	5
						15	54	5

Long bites were mostly associated to the region where the warmer linear heat source was presented (Figure 1). However, when both heat sources were homogeneously heated, it was observed that long bites were distributed over the whole plate (Figure 1). Antennectomized insects showed a similar distribution of long bites, but their frequency was lower than that observed with intact insects.

**Figure 4:**
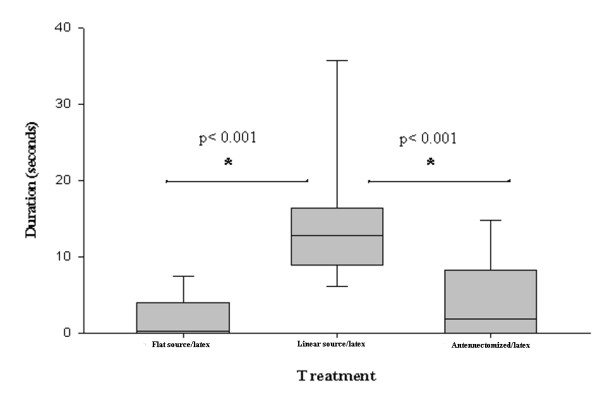
**Mean duration of long bites**. The wire and background were presented at 33°C in the  flat source/latex experiment. The linear heat source was presented at 36°C and the background plate at 33°C in the linear source/latex and antennectomized/latex experiment.

Long bites were significantly longer when a warmer linear heat source was presented, in comparison to those observed with the homogeneously heated source (Figure 4, Mann Whitney, p < 0.001). Similarly, the mean duration of long bites was significantly higher in intact insects than in unilaterally antennectomized insects (Figure 4, Mann Whitney, p < 0.001).

## Discussion

Sensory modalities other than thermo-reception can affect the behavioural sequence displayed by *R. prolixus *after contacting a thermal source. This is evident after comparing the results from experiments with thermal sources covered by a latex film to those from assays with uncovered sources. Insects performed long bites only in the presence of a flexible film covering the warm sources and mostly over the warmer linear source. This strongly suggests that they probed the substratum with their mouthparts in search of a vessel. However, this seems to be triggered only after physical contact of the proboscis has been established on the source and, consequently, after mechanosensory information reporting a flexible surface becomes available. These results suggest that an evaluation of the mechanical properties of the surface over the heat source is accomplished by mechanoreceptors located on the proboscis. Nevertheless, we cannot exclude a role for mechanoreceptors located in other body structures, e.g., antennae and tarsi, in the detection of surface properties. According to our results, temperature would be the main stimulus influencing the choice of a biting location, and mechanical stimuli (e.g. texture and topographic properties of the substratum) would be involved in triggering other phases of the sequence, such as probing with mouthparts. The receptors present on the proboscis of *Triatoma infestans*, the species that acts as main vector of Chagas disease, can be involved in both mechano and thermo-reception during contacts with the surface of the host [6]. Conversely, *R. prolixus *appears to bear mechanoreceptors on the proboscis, but thermoreceptors have not been found on its surface (17).

Interestingly, the proboscis of *T. infestans *presents four morphologically different types of sensilla that may be involved in the perception of mechanical properties of hosts as well as odours, temperature and relative humidity [20].

The ability of *R. prolixus *to locate and, subsequently probe, the warmest sites on the host skin surface has been reported [17]. This capacity would improve the chances of finding a blood vessel, minimizing both the time consumed and the disturbance of the host. The fact that the insects tended to concentrate their long bites over the linear thermal source, reinforces the hypothesis that places with higher temperature probably indicate underlying blood vessels to bugs. It is probable that during these long bites the insects probed and contacted the linear thermal source with their maxillae. This would be possible because the maxillae of *R. prolixus *5^th ^instar nymphs can be protruded between 1-5 mm away from the tip of the proboscis, and therefore they could easily reach the linear heated source from the area most frequently bitten after contact with the latex film [21]. The fact that long bites were only observed after adding a latex cover to a heat source suggests that the multimodal integration of sensory inputs detected by both antennal thermoreceptors and rostral mechanoreceptors is necessary to induce the expression of long bites (Figure 3). Furthermore, the addition of a warmer linear source (Figure 3) was able to induce the maximum proportion of long contacts observed, suggesting that insects tend to increase the expression of long bites when both local thermal gradients and an adequate texture are simultaneously presented to them. This is reinforced by the fact that the duration of long bites was also highest in the presence of a warmer linear source.

The ability of *R. prolixus *to locate the warmest sites on the host skin surface depends on the bilateral integration of thermal inputs by both antennae [17]. Consistently, the total number of contacts of the proboscis performed by the insects in the present study diminished with the removal of one antenna. Nevertheless, a significant reduction was not observed in the proportion of long bites after unilateral antennectomy (Table 1). Seemingly, the removal of one antenna did not affect the probability of triggering the apparent penetration of the film with the mouthparts once the contact with the latex surface was established (Figure 3).

Therefore, the decision of whether to probe the surface appears not to be critically dependant on the bilateral integration of thermal inputs. However, the duration of long bites was shorter in unilaterally antenectomized insects, suggesting that the bilateral integration of thermal information might be relevant for the continuation of long bites.

## Conclusion

This study suggests that the probing process is triggered by the multimodal integration of sensory inputs detected by antennal thermoreceptors and rostral mechanoreceptors and that the heat emitted by blood vessels may be continually evaluated by the antennae of triatomine bugs during probing.

## Competing interests

The authors declare that they have no competing interests.

## Authors' contributions

RAF, MHP and MGL designed the present experiments. RAF carried the experiments. RAF performed statistical analyses. RAF, MHP and MGL contributed to the manuscript redaction and revised it critically. All read and approved the final version of the manuscript.
